# Supplementary material for: Immune engineered extracellular vesicles to modulate T cell activation in the context of type 1 diabetes
Source: Sci Adv. 2023 Jun 2;9(22):eadg1082. doi: 10.1126/sciadv.adg1082 (PMC10765990; doi:10.1126/sciadv.adg1082)
Supplement: Supplementary file 1 — Figs. S1 to S8 Table S1 [file sciadv.adg1082_sm.pdf]

Science Advances
AAAS

Supplementary Materials for

Immune engineered extracellular vesicles to modulate T cell activation in the context of type 1 diabetes

Matthew W. Becker *et al.*

Corresponding author: Edward A. Phelps, ephelps@bme.ufl.edu

Sci. Adv. **9**, eadg1082 (2023)
DOI: 10.1126/sciadv.adg1082

This PDF file includes:

Figs. S1 to S8
Table S1

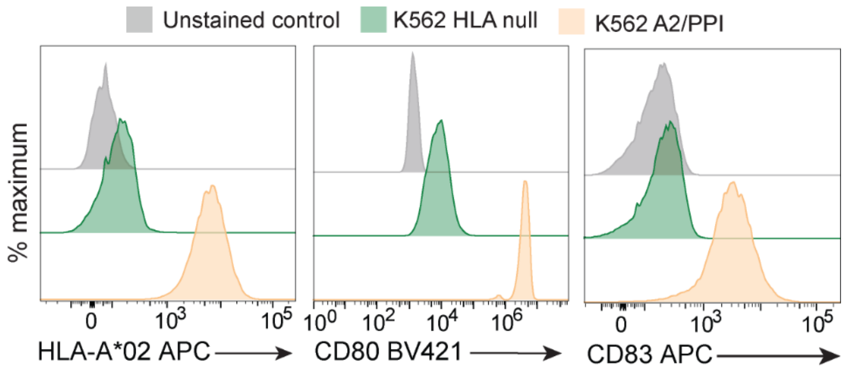

Fig. S1.

Surface expression of HLA-A*02, CD80, and CD83 on K562 cell lines. Flow cytometry staining showing strong expression on K562 A2/PPI cells but not K562 HLA null cells. n=3.

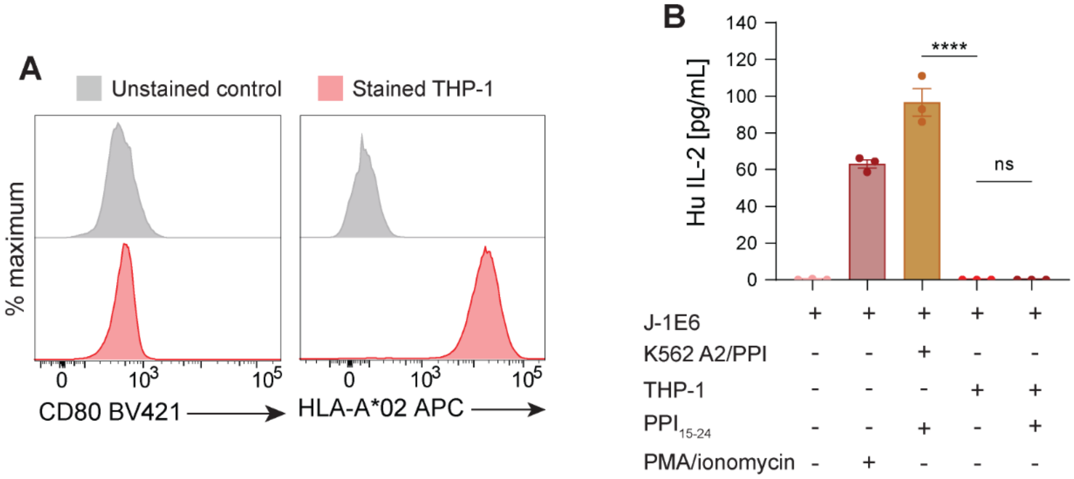

Fig. S2.

Characterization of THP-1 cells and their effects in co-cultures with J-1E6 T cells. (A) Surface expression of CD80 and HLA-A*02 in THP-1 cells. n = 3. (B) IL-2 secretion from J-1E6 T cells co-cultured with either K562 A2/PPI or THP-1 cells. THP-1 cells fail to induce IL-2 secretion from J-1E6 cells even in the presence of exogenous $PPI_{15\text{-}24}$. Representative of 2 independent experiments. Statistical differences for (B) were determined by one-way ANOVA followed by Tukey's multiple comparison test. ns = non-significant, **** $p < 0.0001$.

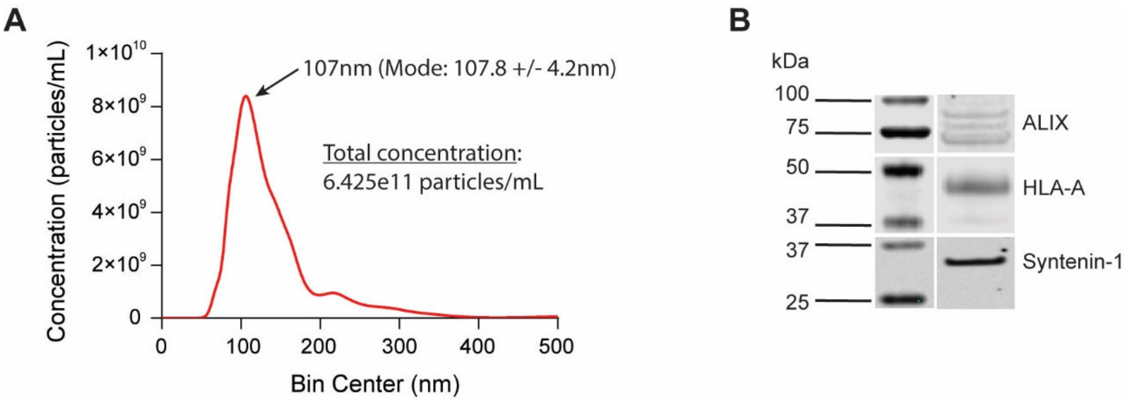

Fig. S3.

Characterization of THP-1 EVs. (A) NTA showing representative particle concentration and size distribution of EVs derived from THP-1 cells. (B) Western blot analysis of EV isolates demonstrating the presence of canonical EV markers. Representative of 4 independent experiments.

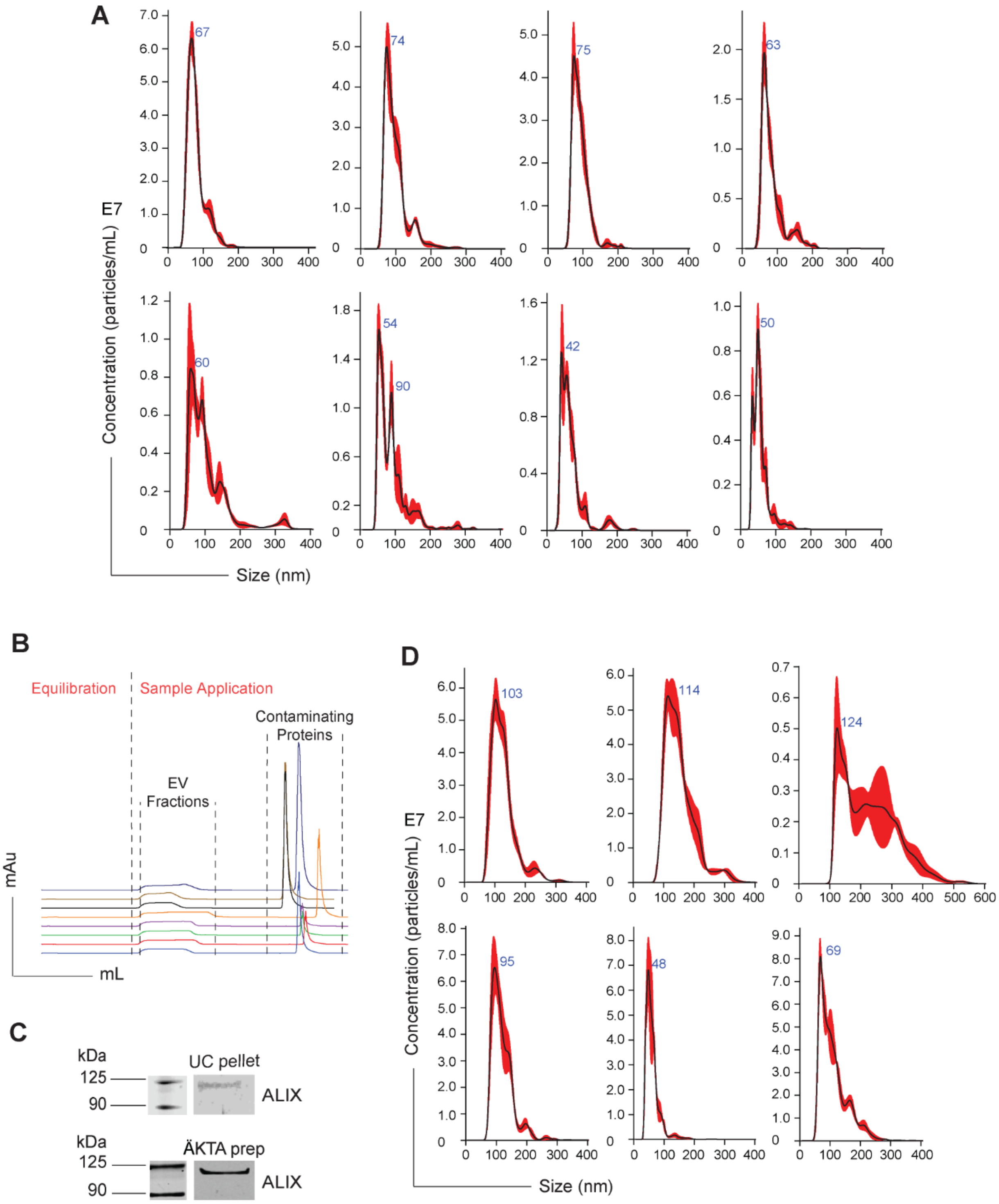
A
E7
Concentration (particles/mL)
Size (nm)
67
74
75
63
60
54
90
42
50
B
Equilibration
Sample Application
Contaminating Proteins
EV Fractions
mAu
mL
C
kDa
UC pellet
125
90
ALIX
ÄKTA prep
D
103
114
124
95
48
69

Fig. S4.

Reproducibility of EV isolations using chromatography combined with ultrafiltration. (A) NTA histograms from eight independent EV isolations using BE-SEC combined with ultrafiltration, showing consistent batch-to-batch particle sizes ranging from 42-75 nm. (B) UV chromatograms from the same eight EV isolations, showing good peak separation of EVs from soluble proteins that is consistent between batches of different starting volumes. (C) Western blot analysis of EV isolates from ultracentrifugation (UC) or BE-SEC combined with ultrafiltration, showing the presence of the canonical small EV marker ALIX in both samples. (D) NTA histograms from six independent EV isolations using ultracentrifugation, showing wider batch-to-batch variability and less consistent particle size distributions compared to BE-SEC isolations.

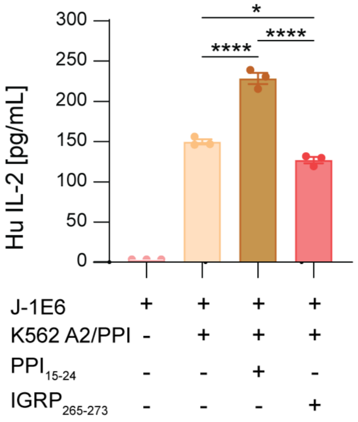

Fig. S5.

Confirming antigen-driven activation of J-1E6 T cells in co-cultures. J-1E6 cells cultured with K562 A2/PPI cells and exogenous PPI_{15-24} have increased IL-2 secretion compared to cultures without exogenous peptide, whereas culture with $IGRP_{265-273}$ peptide does not increase IL-2 secretion. Statistical differences for were determined by one-way ANOVA followed by Tukey's multiple comparisons test. * $p < 0.05$, **** $p < 0.0001$.

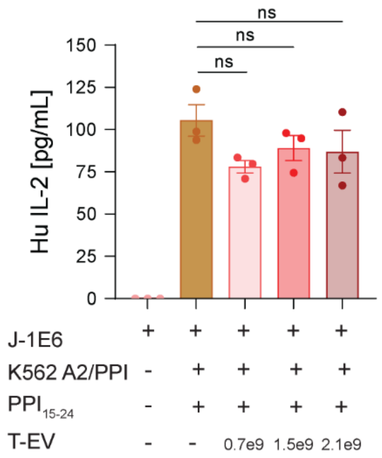

Fig S6.

Effects of THP-1 EVs on J-1E6 T cell activation. IL-2 secretion from J-1E6 T cells after co-culture with K562 A2/PPI cells and increasing amounts of T-EVs, showing no significant change in T cell activation with increasing amounts of EVs. n = 3. Statistical differences were determined by one-way ANOVA followed by Tukey's multiple comparison test. ns = non-significant.

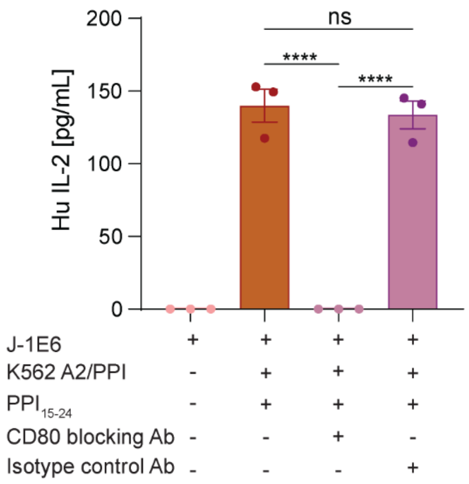

Fig S7.

Confirming the necessity for CD80 co-stimulation in co-cultures with J-1E6 T cells and K562 A2/PPI cells. IL-2 secretion from J-1E6 T cells after co-culture with K562 A2/PPI cells and a CD80 blocking antibody or isotype control, showing complete ablation of the IL-2 response when the blocking antibody is added. n = 3. Statistical differences were determined by one-way ANOVA followed by Tukey's multiple comparison test. ns = non-significant, **** $p < 0.0001$.

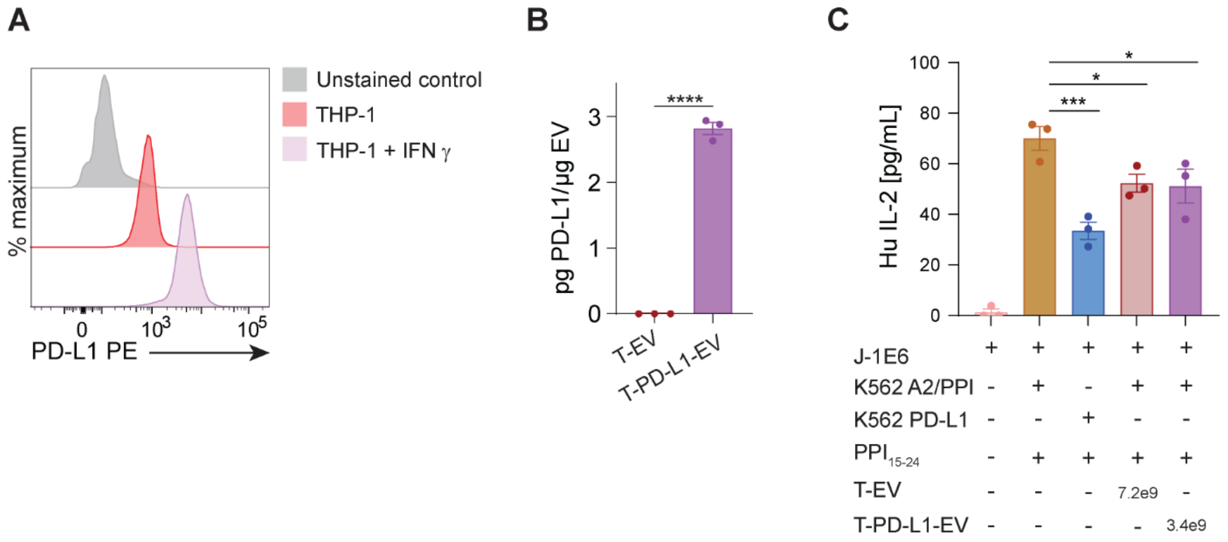

Fig S8.

Driving PD-L1 expression in THP-1 cells and EVs, and effects on J-1E6 T cell activation. (A) Surface expression of PD-L1 in THP-1 cells with or without IFN-γ treatment. $n = 3$. (B) ELISA of PD-L1 on EVs from THP-1 cells with or without IFN-γ treatment compared to total EV protein content. $n = 3$. (C) IL-2 secretion from J-1E6 T cells after co-culture with K562 cells and THP-1 EVs with or without PD-L1, showing slightly decreased T cell activation with excessive amounts of T-EVs or moderate amounts of T-PD-L1-EVs. Statistical differences for (B) were determined by an unpaired, two-tailed t-test. Statistical differences for (C) were determined by one-way ANOVA followed by Tukey's multiple comparison test. * $p < 0.05$, *** $p < 0.001$, **** $p < 0.0001$.

Table S1.
Antibody information. Antibodies used for flow cytometry and western blot analysis.

Antigen	Dilution	Host	Supplier	Catalog number	Clone
CD80	1:50	Mouse	BD	566263	L307.4
HLA-A2	1:50	Mouse	BD	561341	BB7.2
CD83	1:50	Mouse	BD	551073	HB15e
PD-L1	1:50	Mouse	BioLegend	329706	29E.2A3
CD8	1:50	Mouse	BioLegend	301046	RPA-T8
CD69	1:50	Mouse	BioLegend	310916	FN50
PD-1	1:50	Mouse	BD	566460	EH12.1
PD-L1	1:200	Rabbit	Cell Signaling	13694S	E1L3N
ALIX	1:500	Mouse	Abcam	ab117600	3A9
HLA-A	1:1000	Rabbit	Abcam	ab52922	EP1395Y
Syntenin-1	1:500	Rabbit	Abcam	ab133267	EPR8102
beta-actin	1:10000	Mouse	Sigma	A1978	AC-15
